# The impact of COVID-19 pandemic on the health-seeking behaviour of an Asian population with acute respiratory infections in a densely populated community

**DOI:** 10.1186/s12889-021-11200-1

**Published:** 2021-06-23

**Authors:** Hwee Mian Jane Tan, Mui Suan Tan, Zi Ying Chang, Kee Tung Tan, Guan Liang Adrian Ee, Chee Chin David Ng, Ying Khee William Hwang, Yi Ling Eileen Koh, Yan Ping Sarah Low, Ngiap Chuan Tan

**Affiliations:** 1 SingHealth Polyclinics, 167 Jalan Bukit Merah Connection One (Tower 5), #15-10, Singapore, 150167 Singapore; 2 SingHealth Duke-NUS Family Medicine Academic Clinical Program, Singapore, Singapore; 3 National Cancer Centre Singapore, 11 Hospital Crescent, Singapore, 169610 Singapore; 4 SingHealth Duke-NUS Family Oncology Academic Clinical Program, Singapore, Singapore

**Keywords:** COVID-19, Acute respiratory infections, Health-seeking behaviour, Influenza vaccination

## Abstract

**Background:**

The COVID-19 pandemic led to the implementation of various non-pharmaceutical interventions (NPI) as the Singapore government escalated containment efforts from DORSCON Orange to Circuit Breaker. NPI include mandatory mask wearing, hand hygiene, social distancing, and closure of schools and workplaces. Considering the similar mode of transmission of COVID-19 and other pathogens related to acute respiratory infections (ARI), the effects of NPI could possibly lead to decreased ARI attendances in the community. This study aims to determine the year-on-year and weekly changes of ARI attendances across a cluster of polyclinics following the implementation of NPI.

**Methods:**

The effect of the nation-wide measures on the health-seeking behaviour of the study population was examined over three periods: (1) 9 weeks prior to the start of Circuit Breaker (DORSCON Orange period), (2) 8 weeks during the Circuit Breaker, and (3) 9 weeks after easing of Circuit Breaker. Data on ARI attendances for the corresponding periods in 2019 were also extracted for comparison and to assess the seasonal variations of ARI. The average weekly workday ARI attendances were compared with those of the preceding week using Wilcoxon signed rank test.

**Results:**

ARI attendances dropped steadily throughout the study period and were 50–80% lower than in 2019 since Circuit Breaker. They remained low even after Circuit Breaker ended. Positivity rate for influenza-like illnesses samples in the community was 0.0% from the last week of Circuit Breaker to end of study period.

**Conclusions:**

NPI and public education measures during DORSCON Orange and Circuit Breaker periods appear to be associated with the health-seeking behaviour of the public. Changing levels of perceived susceptibility, severity, benefits and barriers, and widespread visual cues based on the Health Belief Model may account for this change. Understanding the impact of NPI and shifts in the public’s health-seeking behaviour will be relevant and helpful in the planning of future pandemic responses.

## Background

The novel coronavirus 2 (SARS-CoV-2) or COVID-19 infection was declared a pandemic by the World Health Organization (WHO) on 11 March 2020, and has infected more than 45 million people globally as of end October 2020 [1]. Current evidence shows that the COVID-19 virus is mainly transmitted via respiratory droplets and contact routes such as direct contact with infected people and indirect contact with fomites [2–6]. Preventive measures to reduce its transmission include wearing of face masks, good hand hygiene and isolation of infected persons [6, 7]. The escalation of the pandemic has resulted in many nations enacting emergency health policies and national-wide measures to decelerate the spread of the virus.

The COVID-19 pandemic led to the establishment of a multi-ministry taskforce by the Singapore government to manage the local outbreak. The taskforce leverages on the Disease Outbreak Response System Condition (DORSCON) to guide the measures to be implemented in the community and provide advice to the public [ 8]. It raised the DORSCON to Yellow on 21 January 2020 and to Orange level on 7 February 2020. A set of preventive measures was mandated as a “Circuit Breaker” (CB) to curb the transmission of COVID-19 within the community [9]. These CB measures were implemented from 7 April to 1 June 2020, and COVID-19 (Temporary Measures) Act 2020 was gazetted to ensure strict enforcement of the measures [10]. The CB measures include social distancing, working from home for employees, and full home-based learning for students. Free reusable masks were distributed to all residents. Recreation venues, attractions and places of worship were closed, and older persons were advised to stay home. Members of the public were advised to be socially responsible by practising good hand hygiene, wearing masks when going out, and consult a doctor if they were sick. Violation of these measures were subjected to penalties. People with symptoms of Acute Respiratory Infections (ARI) were given mandatory five-day sick leave to recover at home, and were not allowed to leave their homes except to seek medical attention. Those who failed to comply were liable to a fine of up to $10,000, or imprisonment up to six months, or both, under the Infectious Diseases Act [11].

Singapore reported its first case of COVID-19 on 23 January 2020, and has more than 50,000 cases as of 31 October 2020 [12]. It is densely populated with 5.7 million people living on the 721.5 km^2^ tropical island-state. Over 70% of its multi-ethnic Asian population live in close proximity within public high-rise housing estates [13]. Pathogens such as influenza, parainfluenza, pneumococcus causing ARI are endemic. Like the COVID-19 virus, they are similarly transmitted from person-to-person via respiratory droplets, direct contact and fomites [14].

These ARIs are commonly managed by primary healthcare providers in Singapore. The 20 public primary care clinics (polyclinics) and about 1700 private general practitioner (GP) clinics are the major local primary healthcare providers [15]. Data from the Singapore Ministry of Health (MOH) showed over 153,000 ARI attendances in the polyclinics annually over the past three years from 2017 to 2019 respectively [16]. ARIs are among the top 4 conditions seen at the polyclinics, making up 8.6–9.4% of polyclinic attendances in the past three years [17].

Strict enforcement of nation-wide measures to contain the COVID-19 outbreak are postulated to affect the incidences of ARI. Non-pharmaceutical interventions (NPI) in the community, such as social distancing, have shown to be effective in reducing influenza transmission in East Asia [18–21]. Preventive measures such as up-to-date influenza vaccinations will also mitigate the ARI risk in the community. Soo RJJ et al. reported that influenza activity had declined in Singapore in the current year 2020 based on routine sentinel surveillance data on influenza-like infections from a national network of primary care clinics and the National Public Health Laboratory, suggesting that the measures taken for COVID-19 were effective in reducing the local spread of other respiratory diseases [22]. Such estimates of influenza were based on sentinel surveillance and therefore healthcare seeking behaviour. Hence the reduction in influenza incidence is possibly more a measure of reduced healthcare seeking and not necessarily reduced transmission of influenza due to NPIs. Considering the similar mode of transmission of COVID-19 and other pathogens related to ARI, the effect of NPI and collective CB measures are postulated to decrease the ARI attendances in the community in Singapore.

### Study aim

This study aims to determine the year-on-year and weekly changes of ARI attendances across a cluster of polyclinics following the implementation of NPI.

## Method

### Study site

SingHealth Polyclinics (SHP) serve an estimated 1.3 million residents, which constitutes 23% of Singapore’s 5.7 million population, living in the eastern region of the island state [15]. These polyclinics serve as “one-stop” healthcare centres providing accessible and affordable primary healthcare services to the local residents, including influenza and pneumococcal vaccinations. SHP manage about 6400 patients daily during each workday in its network of 8 polyclinics [23]. In 2019, an average of 5471 patients attended these polyclinics for ARI per week based on disease coding in the electronic medical records system [24].

### Study period

The effect of the nation-wide measures on the health-seeking behaviour of the study population was examined over three periods [1]: 9 weeks prior to the start of Circuit Breaker (corresponding to DORSCON Orange period) [2], 8 weeks during the Circuit Breaker (7 April to 1 June 2020), and [3] 9 weeks after easing of Circuit Breaker (2 June to 1 August 2020). The public was allowed to seek medical attention, including ARI, throughout the observation period. Data for the corresponding periods in the preceding year was also extracted for comparison and to assess the seasonal variations of ARI.

### Study population

The study population consisted of all patients who attended SHP for ARI during the observation period. This included Singapore citizens, permanent residents and non-residents of all ages who may either walk-in or make a prior appointment to be seen at any of the SHP clinics. Patients may self-present or be referred by medical practitioners in both public and private practice.

### Definition of case

ARI attendance was defined as a visit by a patient to any branch of SHP for the diagnoses of acute bronchitis, influenza-like illness (ILI), upper respiratory tract infection (URTI) and pneumonia. These diagnoses were coded by the attending doctor into the SHP electronic medical records system, Sunrise Clinical Manager (SCM). The codes originate from the International Classification of Diseases (ICD-10) codes J20.9, J10.1, J06.9 and J18.9 respectively.

### Database and data extraction

The clinical information and records documented by the polyclinic staff in the SCM and OAS are channelled into the institution’s data warehouse, Enterprise Health Intelligence System (eHIntS). eHIntS is an enterprise business intelligence repository integrating clinical information, business workload and finance for reporting and healthcare analysis. The study data was extracted from eHIntS based on the four stipulated ARI diagnoses tagged to their corresponding ICD-10 codes within the observation period.

### Data

Two main datasets were extracted [1]: the total weekly ARI attendances at the 8 polyclinics, which were grouped according to age, gender, ethnicity and respective clinic; and [2] the total weekly attendances for each of the 4 ARI diagnoses at all 8 SHP clinics. The age groups were specified as child (0–16 years old), adult (17–64 years old) and older adult (≥65 years old). This was performed by setting filters in eHIntS for time periods, diagnosis codes, age limits, race, and gender. The polyclinics are operational for half a day on Saturdays and closed on Sundays and public holidays. The average weekly ARI attendances were computed based on number of workdays for the specific week.

### Data management

The data was extracted and processed by the Health Information Department. The Principal Investigator documented the data extraction algorithm to allow for data re-examination if necessary. The data was recorded in Microsoft Excel spreadsheets by the PI and backup copies maintained by the co-investigators in password-protected computers. Data would be archived for 7 years as per local research ethics guidelines and policy.

### Statistical analysis

**Fig. 1:**
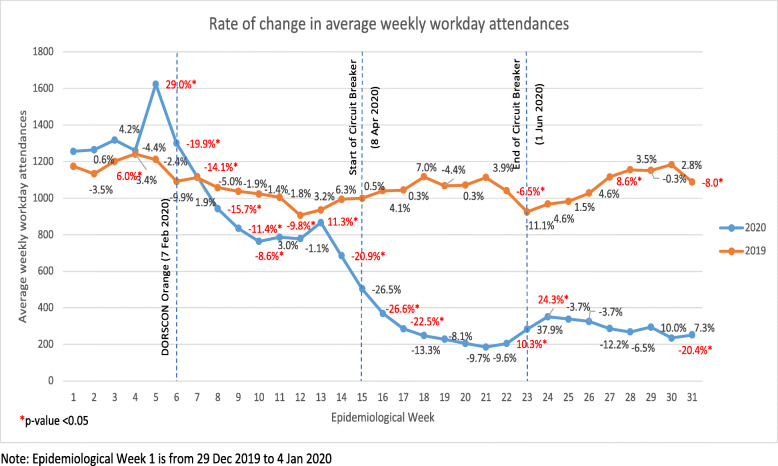
Rate of change in average weekly workday attendances

**Fig. 2:**
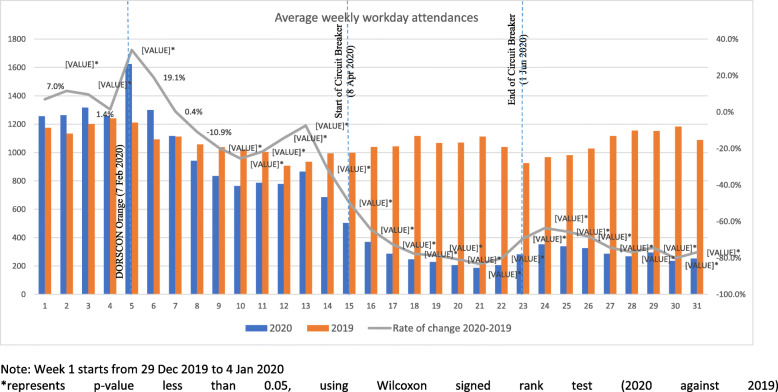
Comparison of average weekly workday attendances in 2020 with 2019. Note: Week 1 starts from 29 Dec 2019 to 4 Jan 2020. *represents *p*-value less than 0.05, using Wilcoxon signed rank test (2020 against 2019)

The average weekly workday ARI attendances from 2019 to 2020 were presented in Fig. 1. The difference between the average weekly attendances compared with those of the preceding week was assessed using Wilcoxon signed rank test. The rate of change of average weekly workday attendances was derived from taking [Attendances in Week n – Attendances in Week (n-1)]/ Attendance in Week (n-1). With the NPI in place in 2020, comparison of weekly workday attendances in 2020 with those in the absence of NPI in 2019 was performed using Wilcoxon signed rank test (Fig. 2). The rate of change from 2019 to 2020 was computed using the difference between average weekly workday attendances in 2020 and 2019 divided by average weekly workday attendances in 2019. All analyses and charts were performed and plotted using IBM SPSS 25.0 and Excel. A *p*-value of less than 0.05 is considered statistically significant.

## Results

The results are presented chronologically across the three stipulated periods.

**a) Average Workday Total ARI Attendances Per Week.**

### DORSCON Orange

The ARI attendances were generally higher in early 2020 than 2019. There was a significant peak just before the start of DORSCON Orange with an average workday attendances of 1624 per week. It reflected the greatest rate of increase of 29.0% from the preceding week. This was followed by a steep decline of 19.9% from the first week of DORSCON Orange period. Henceforth, there was a steady decline of average weekly workday attendances and also when compared to 2019, reaching a lowest average weekly workday attendances of 685 at week 14. There was a brief period of statistically significant rise of 11.3% in ARI attendances from week 12 to 13.

### Circuit breaker

CB period saw a further decline in the ARI attendances. There was a significant drop in average workday ARI attendances per week by 26.6% after the first week of CB, and by 22.5% after the second week of CB.

Compared to the corresponding period in 2019, which had a consistent average workday attendance per week of 1061, the same period in year 2020 saw a steady weekly drop in ARI attendances from a weekly workday average of 504 in week 15 (start of CB) to a lowest of 186 in week 21. Towards the end of CB, there was a gradual rise in the ARI attendances, from an average of 186 in week 21 to 283 in week 23. This was reflected as a significant increase of 10.3% at week 23, which corresponded to the end of CB.

### End of circuit breaker

The only statistically significant increase in average weekly workday ARI attendances of 23.4% was seen in the first week after the end of CB. This was then followed by a 4-week period of non-statistically significant decline in ARI attendances. Week 30 showed a statistically significant decline in ARI attendances of 20.4%.

The corresponding period in 2019 showed much higher ARI attendances. The average workday ARI attendances per week in 2019 showed a continuous increase with the only exceptions being weeks 28 and 31 having statistically significant declines of 0.3 and 8% respectively.

The average weekly workday ARI attendances in 2020 were statistically compared with those of the same epidemiological weeks in 2019 as shown in Fig. 2. Following the implementation of DORSCON Orange, there was a statistically significant decline in the rate of change in ARI attendances ranging from 7.4 to 31.1% in ARI attendances compared to 2019. The rate of change in ARI attendances showed an even more marked and steady decline throughout the CB period, ranging from a drop of 49.6 to 83.3%. After easing of CB, ARI attendances in 2020 were still much lower than in 2019, and the rate of change was rather consistent as during CB and ranged from 63.7 to 80.2%.

**b) Average Workday ARI Attendances Per Week By Age Groups.**

**Fig. 3:**
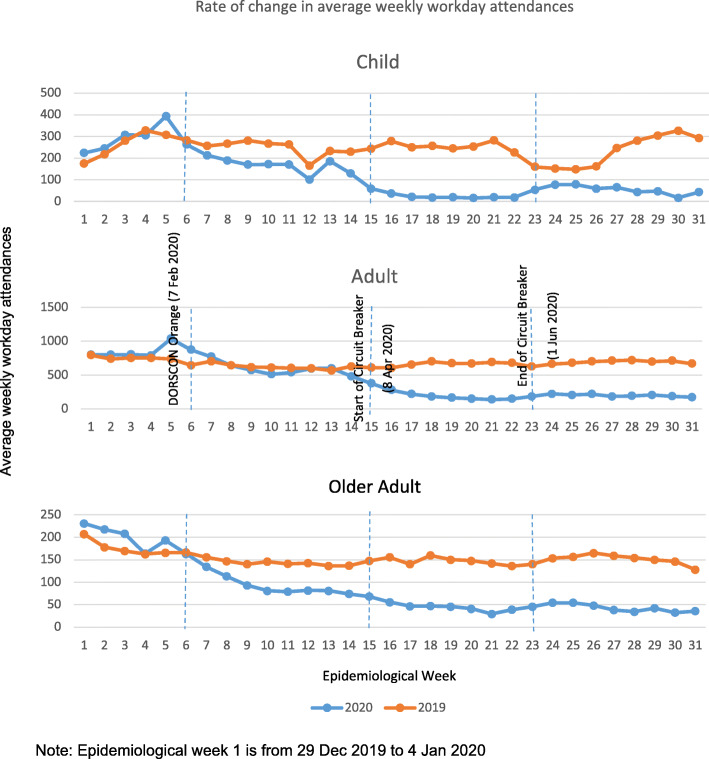
Rate of change in average weekly workday attendances by age groups

Average weekly workday attendances were then stratified according to the 3 specified age groups as shown in Fig. 3. The decrease in average workday ARI attendances per week was seen in all 3 age groups during the CB period, with the greatest drop seen amongst the children, from an average of 247 in 2019 to 121 in 2020.

### Child ARI attendances

Prior to DORSCON Orange, an average 295 and 262 workday paediatric attendances for ARI for 2019 and 2020 respectively were observed. However, when DORSCON Orange measures were implemented, such attendances declined steadily to 177 in 2020 compared to 249 in 2019. During the CB, the attendances declined significantly further to an average weekly workday attendance of 26 compared to 254 in 2019. This attendance increased slightly with easing of CB measures from week 23 to 54, but was still lower than the mean attendances of 230 in 2019.

### Adult ARI attendances

For epidemiological weeks 1–5, the average workday adult ARI attendances per week in 2020 was higher at 847 compared to 754 in 2019. During the DORSCON Orange period, the average workday child ARI attendances for adults were comparable, at 620 in 2020 compared to 624 in 2019. During the CB, the average workday adult ARI attendance per week in 2020 declined to 207 compared to 660 in 2019 during epidemiological weeks 15–22. This reflected an approximate 70% reduction in attendance.

### Older adult ARI attendances

For older adults, the average workday attendances per week in 2020 declined by half (51%) during the DORSCON Orange period and by a further 53% to 47 during the CB. This was in contrast to 2019 when the average workday attendance per week remained fairly constant at approximately 150.

**c) Average Workday ARI Attendances Per Week By Diagnosis.**

**Fig. 4:**
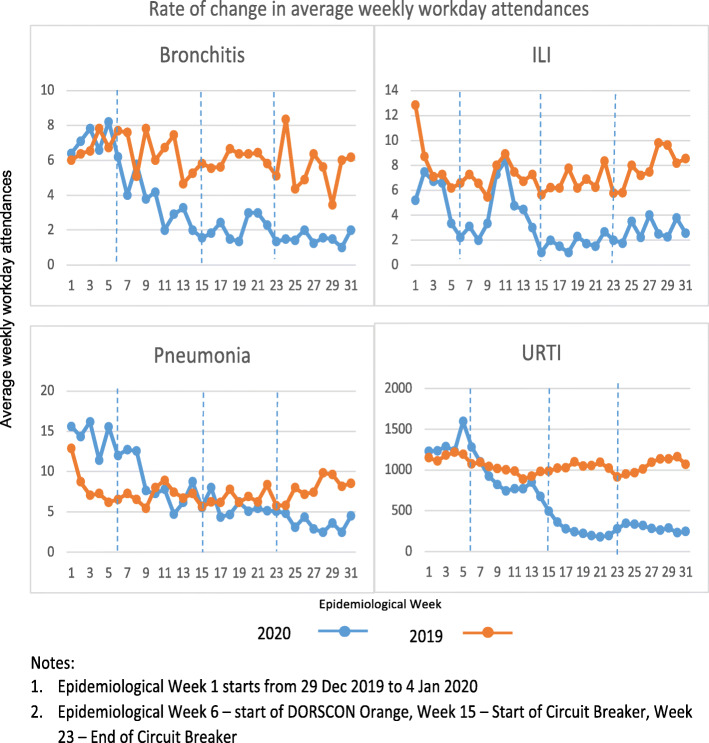
Rate of change in average weekly workday attendances by diagnosis

Average weekly workday attendances were also stratified according to the 4 specified ARI diagnoses as shown in Fig. 4.

### Bronchitis

Average workday bronchitis attendances per week showed a steady decline throughout DORSCON Orange period. The rate of decline was steepest during DORSCON Orange, as reflected by a drop of 66.7% during this period. Subsequently, the attendances generally levelled off at this low level during CB period. When CB period ended, the average weekly attendances decreased by 69.0%, as compared to the corresponding period in 2019.

### Ili

Average workday ILI attendances per week showed 2 peaks in attendances in 2020. The first peak occurred at epidemiological week 2, which coincided with one of the bimodal increases in influenza incidence from November to January in Singapore. It corresponded approximately to the influenza season in the Northern hemisphere. The second peak in average workday ILI attendances per week was unexpected. It occurred approximately 6 weeks after DORSCON Orange was declared. The increase of 86.7% in 2020 was higher than the peak in 2019.

### Pneumonia

Prior to DORSCON Orange period, the average weekly attendances for pneumonia in 2020 was higher than similar period in 2019. There was an average weekly attendance of 15 in the first 5 weeks of 2020 compared to 8 in 2019. The sharpest decline for pneumonia related attendances was noted during DORSCON Orange and levelled off during CB. Subsequently there was a gentle decline in the post Circuit Breaker period, which was a contrast to the increase in attendances seen in 2019 for this period.

### URTI

There was a spike in average weekly attendances just before the implementation of DORSCON Orange in 2020, as compared to the same period in 2019. This was followed by a decline of 27.9% during the first half of DORSCON Orange period. URTI attendances continued to drop further during the CB period, accounting for an average decline of 74% during this period. It remained at this low level even after the CB period ended.

## Discussion

The average weekly workday ARI attendances showed a steady decline throughout both DORSCON Orange and CB periods, but with a slight transient increase of around 10–24% at the end of each period. Even after easing of CB, the ARI attendances remained lower than those in 2019 by 62.6–81.5**%.**

Our findings corroborated with national data from Singapore’s MOH which reported weekly polyclinic attendances for ARI. The overall positivity rate for influenza among ILI samples in the community was 0.0% since July 2020. 320 out of a sample of 652 people tested positive for influenza in January 2020 compared to 1 positive case each in April and May 2020 [16]. It suggests that the NPI during CB have invariably impacted on the healthcare seeking behaviour of the public.

School and workplace closures are major NPI. A systematic review by Rashid et al. [25] suggests that such closures can result in moderate and modest reduction in influenza transmission and deferred peak of an epidemic. Ajelli et al. [26] alluded that school closure over the weekends significantly affected the pattern of transmission. Cauchemez et al. [27] also reported strong association between-place interactions with back- and-forth waves of influenza transmission between the school, the community, and the households.

**Fig. 5:**
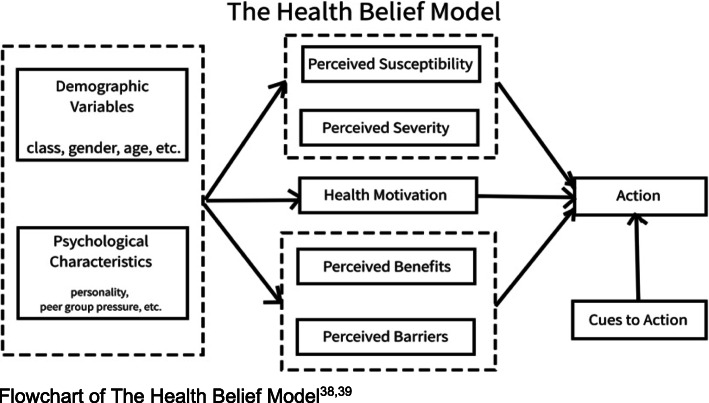
Health Belief Model. Flowchart of The Health Belief Model [28, 29]

The Health Belief Model has provided a theoretical framework to understand why people wore mask during the SARS pandemic [28]. As depicted in Fig. 5, the model can be used to frame the public’s health-seeking behaviour change in the current pandemic.

In line with the Health Belief Model [29, 30], the escalation to DORSCON Orange and CB increased the public’s “perceived susceptibility” and “perceived severity” of COVID-19 in the community, and also served as “cues to action” to take on social distancing measures, such as staying home as much as possible. Stricter social distancing measures and associated punitive measures for violations were explicit visual cues when they were implemented at the end of week 13.

The public’s “perceived barriers” to seeking medical attention for mild ARI could have outweighed their “perceived benefits” of visiting a healthcare establishment. “Perceived barriers” such as risk of transmission of COVID-19 when out of their homes, issuance of mandatory 5-day sick leave and fear of having to undergo a COVID-19 nasopharyngeal swab test greatly outweighed the ‘“perceived benefits” of a medical attendance. Mild ARI could easily be self-medicated at home. Closure of non-essential workplaces and all schools negated the need to obtain sick leave when unwell.

Individual NPI such as mandatory mask wearing, hand hygiene and respiratory vaccinations have been associated with decreased ARI attendances throughout and post CB. However, a systematic review by Wang et al. [31] found that surgical mask usage had a non-significant protective effect in reducing the risk of ARI among asymptomatic individuals in non-healthcare settings. This contrasts with a review by Jefferson et al. [32], which found face mask to be the best performing intervention compared with other physical NPI studied across different populations and settings. Another systematic review by Cowling et al. [7] reveals evidence that wearing of masks or respirators during illness protect others, and publicity of its usage during illness may help to reduce influenza virus transmission. Chou et al. [33] reported that mask protection against respiratory infection prevention was more effective in healthcare than community settings. However, its efficacy can be highly variable. Hence it is difficult to attribute the decline in ARI attendances to decreased transmission of ARI from mask wearing alone. 

The evidence for hand hygiene, another individual NPI in curbing ARI transmission, is equivocal. Warren-Gash et al. [34] in their systematic review shows the greatest mitigating effect of hand hygiene only in two studies in low to middle-income settings. Similarly, a Swedish population-based study by Merk et al. [35] suggests that increased adult perception of adequacy of hand-washing may not significantly reduce the ARI risk.

The evolving data from a cluster of polyclinics which serve at least a third of the Singapore population constitutes a strength in the study. Our study was based on aggregated attendances from all ages and demographics within the study period, thereby minimising sample bias. Data was compared among the various phases of pandemic containment in 2020, as well as year-on-year with the preceding year, to minimise effect of seasonal variation. Implementation and compliance with NPI of this extent is not common, but was largely achievable during our study period due to the communitarian culture and trust in the leadership of the nation. The study hence provided a rare opportunity to evaluate the impact of a large array of NPI implemented as part of nationwide pandemic containment efforts by the government.

The study is limited to attendances in public primary care clinics. ARI attendances at the private GP clinics were excluded. The data were confined to 9 weeks of ARI attendances before and after the lockdown in 2020 and 2019. However, these were deemed to be sufficient to account for normal variations in weekly ARI attendances. Data on ARI attendances by individuals despite prior influenza and pneumococcal vaccinations were not available in this study.

This study highlighted the association of community NPI, respiratory vaccinations, and shifts in the public’s health-seeking behaviour on decreasing ARI attendances during a respiratory infectious disease pandemic. This was congruent with a study by Noh et al. [36] who reported the association of extensive application of NPI in response to COVID-19 and reduced influenza epidemic in South Korea. By engaging and educating the community across all age groups on the modes of transmission and methods to decrease ARI transmission from henceforth, the public’s knowledge of and compliance with NPI and appropriate vaccinations are expected to improve. Such measures should be integrated as key components in any emerging infectious disease outbreak preparedness action plan.

## Conclusion

NPI and public education measures during DORSCON Orange and CB periods appear to be associated with overall decreased ARI attendances in primary care clinics during the stipulated observation period. Changing levels of perceived susceptibility, severity, benefits and barriers, and widespread visual cues based on the Health Belief Model may account for the change in health-seeking behaviour. Understanding the impact of NPI on shifts in the public’s health-seeking behaviour and ARI transmission will be relevant and helpful in the planning of future pandemic responses.

## Data Availability

The datasets generated and/or analysed during the study are not publicly available but are available from the corresponding author on reasonable request.

## Abbreviations

ARI: Acute Respiratory Infections
CB: Circuit Breaker
CDC: Centres for Disease Control and Prevention
CIRB: SingHealth Centralised Institutional Review Board
DORSCON: Disease Outbreak Response System Condition
eHIntS: Enterprise Health Intelligence System
GP: General Practitioner
HBL: Home Based Learning
MCO: Movement Control Order
MOH: Ministry of Health
NPI: Non-pharmaceutical interventions
OAS: Outpatient Administration System
SCM: Sunrise Clinical Manager
SHN: Stay Home Notice
SHP: SingHealth Polyclinics
URTI: upper respiratory tract infection
WFH: Work From Home
WHO: World Health Organisation
